# Advances in molecular characterization of pediatric acute megakaryoblastic leukemia not associated with Down syndrome; impact on therapy development

**DOI:** 10.3389/fcell.2023.1170622

**Published:** 2023-06-01

**Authors:** Jixia Li, Maggie L. Kalev‐Zylinska

**Affiliations:** ^1^ Blood and Cancer Biology Laboratory, Department of Molecular Medicine and Pathology, University of Auckland, Auckland, New Zealand; ^2^ Department of Laboratory Medicine, School of Medicine, Foshan University, Foshan, China; ^3^ Haematology Laboratory, Department of Pathology and Laboratory Medicine, Auckland City Hospital, Auckland, New Zealand

**Keywords:** acute myeloid leukemia, acute megakaryoblastic leukemia (AMKL), non-Down syndrome AMKL, chimeric fusions, cytogenetics, genomics, transcriptomics, therapeutic targets

## Abstract

Acute megakaryoblastic leukemia (AMKL) is a rare subtype of acute myeloid leukemia (AML) in which leukemic blasts have megakaryocytic features. AMKL makes up 4%–15% of newly diagnosed pediatric AML, typically affecting young children (less than 2 years old). AMKL associated with Down syndrome (DS) shows *GATA1* mutations and has a favorable prognosis. In contrast, AMKL in children without DS is often associated with recurrent and mutually exclusive chimeric fusion genes and has an unfavorable prognosis. This review mainly summarizes the unique features of pediatric non-DS AMKL and highlights the development of novel therapies for high-risk patients. Due to the rarity of pediatric AMKL, large-scale multi-center studies are needed to progress molecular characterization of this disease. Better disease models are also required to test leukemogenic mechanisms and emerging therapies.

## 1 Introduction

Acute megakaryoblastic leukemia (AMKL) is a rare but life-threatening hematological malignancy characterized by clonal megakaryoblastic proliferation and impaired differentiation of these cells. AMKL was first described in 1931 and included in the French-American-British (FAB) classification as acute myeloid leukemia (AML) the M7 subtype (De Marchi et al., 2019). In the 2016 World Health Organization (WHO) revision of myeloid neoplasms, AMKL in Down syndrome (DS) (also named as myeloid leukemia associated with DS, ML-DS) was included in myeloid proliferations associated with DS, while AMKL in non-Down syndrome (non-DS) patients was included in the category of acute myeloid leukemia not otherwise specified (AML NOS) (Arber et al., 2016). This categorization remains similar in the latest 2022 WHO and International Consensus classifications (ICC) (Arber et al., 2022; Khoury et al., 2022). However, cases with *KMT2A* (lysine methyltransferase 2A) and *NUP98* (nucleoporin 98) rearrangements now form independent subgroups of AML with defining genetic abnormalities (that also include other non-AMKL phenotypes), and cases with *CBFA2T3* (CBFA2/RUNX1 partner transcriptional co-repressor 3)*::GLIS2* (GLIS family zinc finger 2) and *RBM15* (RNA binding motif protein 15)*::MKL1* (megakaryoblastic leukemia 1) fusions belong to a subgroup of AML with other defined genetic alterations. Other cases of AMKL lacking defined genetic alterations remain defined by differentiation, which requires blasts to express at least one of the platelet glycoproteins: CD41 (glycoprotein IIb), CD61 (glycoprotein IIIa), or CD42b (glycoprotein Ib). Myeloid proliferations associated with DS form a subtype of myeloid neoplasms associated with germline predisposition (Arber et al., 2022; Khoury et al., 2022).

AMKL has a bimodal age distribution, so it can be grouped into adult and pediatric. AMKL is very rare in adults accounting for only 1% of all AML (Wen et al., 2011), but in children, AMKL makes up 4%–15% of newly diagnosed AML cases (de Rooij et al., 2017). AMKL is most frequent in young children with DS (median age at diagnosis 1–1.8 years), accounting for the vast majority of AML in DS (∼70%) (Bhatnagar et al., 2016; Masetti et al., 2019b). By contrast, in non-DS patients, AMKL comprises 5%–10% of AML with a slightly older median age at diagnosis ranging from 1.6 to 1.8 years (de Rooij et al., 2016; Maarouf et al., 2019). Genomic studies highlighted the heterogeneity of pediatric AMKL with the specific alterations characterizing different disease subgroups. ML-DS is driven by overexpressed genes and micro-RNAs located on chromosome 21, *GATA1* (GATA Binding Protein 1) mutations, and a range of other somatic mutations, in particular those affecting cohesin molecules, signaling pathways, epigenetic regulators, and hematopoietic transcription factors (Boucher et al., 2021; de Castro et al., 2021; Grimm et al., 2021). In contrast, non-DS AMKL is characterized by recurrent chromosomal translocations, complex karyotype, and DNA copy number abnormalities (de Rooij et al., 2017). Children with non-DS AMKL show more heterogeneity and more chromosomal abnormalities than ML-DS. Strikingly, recurrent oncogene fusions are found in more than 70% of non-DS pediatric AMKL, including *CBFA2T3::GLIS2*, *KMT2A* rearrangements, *NUP98::KDM5A* (lysine-specific demethylase 5A), *RBM15::MKL1*, and *HOX* (homeobox) gene rearrangements (de Rooij et al., 2016; de Rooij et al., 2017). For completeness, AMKL in adults is more often secondary, preceded by an antecedent hematologic disorder or exposure to chemotherapy or radiotherapy (Dastugue et al., 2002). Adults with AMKL have different cytogenetic and molecular features compared to pediatric patients, showing a diversity of chromosomal abnormalities, the lack of recurrent genetic fusions, and the presence of highly recurrent mutations in genes encoding TP53 (tumor protein p53), cohesins, splicing factors, and epigenetic regulators such as ASXL (additional sex combs-like), DNMT3A (DNA methyltransferase 3 alpha), and TET2 (tet methylcytosine dioxygenase 2) (Dastugue et al., 2002; de Rooij et al., 2017). Cytogenetic and molecular aberrations influence the outcomes of pediatric AMKL subgroups. ML-DS has an excellent prognosis even with low-dose induction chemotherapy, while the outcomes of non-DS AMKL remain unsatisfactory despite improved diagnostics, intensified treatment protocols, and advanced supportive care (de Rooij et al., 2017; Wang et al., 2021). Non-DS children carrying *CBFA2T3::GLIS2*, *KMT2A* rearrangements and *NUP98*::*KDM5A* have a very poor prognosis (de Rooij et al., 2016; de Rooij et al., 2017). Molecular features of ML-DS have been recently reviewed, including by our group (Boucher et al., 2021; de Castro et al., 2021; Grimm et al., 2021; Li and Kalev-Zylinska, 2022). Here, we provide an updated summary of molecular abnormalities and emerging novel therapeutic strategies in pediatric non-DS AMKL.

## 2 Molecular characterization of pediatric non-DS AMKL

**FIGURE 1:**
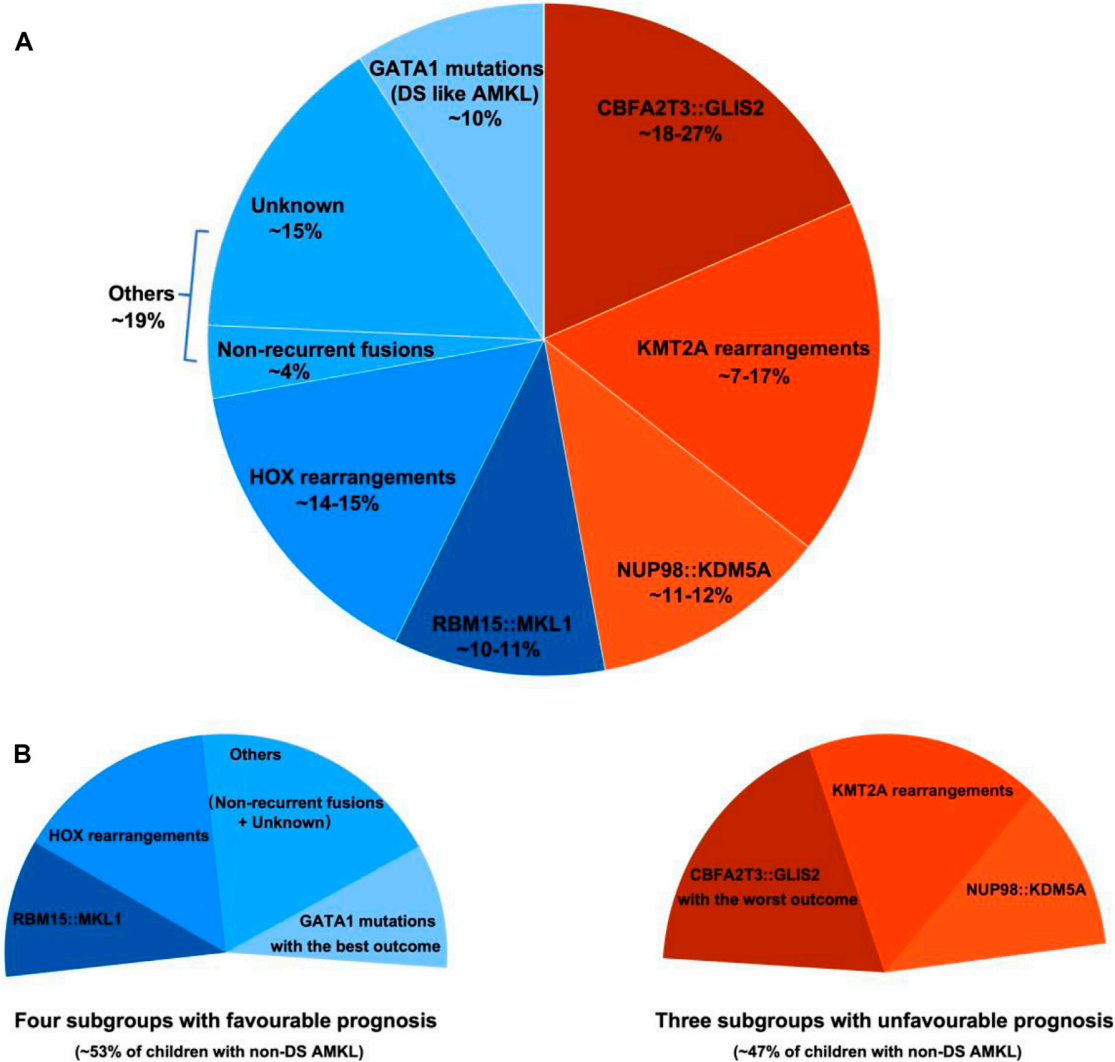
Distribution of distinct molecular subgroups and their prognostic impact in pediatric non-DS AMKL. **(A)** Distribution of distinct molecular subgroups in pediatric non-DS AMKL. Pediatric non-DS AMKL is characterized by recurrent and mutually exclusive chromosomal abnormalities, primarily including *CBFA2T3::GLIS2* (18%–27%), *KMT2A* rearrangements (*KMT2A*r) (7%–17%), *NUP98::KDM5A* (11%–12%), *RBM15::MKL1* (10%–11%), and *HOX* rearrangements (*HOX*r) (14%–15%) (de Rooij et al., 2016; de Rooij et al., 2017; Hara et al., 2017; Lalonde et al., 2021). Further 4% of patients have non-recurrent fusions engaging hematopoietic transcription factors and epigenetic regulators (de Rooij et al., 2017). Other cases do not have known oncogenic fusions, but *GATA1* mutations are detected in about half of this population (overall found in approximately 10% of pediatric non-DS AMKL) (de Rooij et al., 2017). **(B)** Prognosis of distinct molecular subgroups in pediatric non-DS AMKL. Based on molecular aberrations, three subgroups (*CBFA2T3::GLIS2*, *KMT2A*r, and *NUP98::KDM5A*) have an unfavorable prognosis, amounting to nearly half of patients; other three subgroups (*RBM15::MKL1*, *HOX*r, non-recurrent fusions and unknown drivers) have an intermediate prognosis, accounting for approximately 40% of patients; and patients with *GATA1* mutations represent an excellent prognostic group, making up for roughly 10% of children with non-DS AMKL (de Rooij et al., 2017). Among the unfavorable subsets, *CBFA2T3::GLIS2* has the worst outcome, with the 5-year overall survival rate being 14% (de Rooij et al., 2017). In contrast, DS-like AMKL has the best outcome, with the 5-year overall survival rate being 100% (de Rooij et al., 2017).

Pediatric non-DS AMKL (also referred to in this review as non-DS AMKL for simplicity) is a highly molecularly heterogeneous disease characterized by recurrent and mutually exclusive genetic aberrations, including *CBFA2T3*::*GLIS2* (18%–27%), *KMT2A* rearrangements (*KMT2A*r) (7%–17%), *NUP98*::*KDM5A* (11%–12%), *RBM15*::*MKL1* (10%–11%), and *HOX* rearrangements (*HOX*r) (14%–15%) (Figure 1A) (de Rooij et al., 2016; de Rooij et al., 2017; Masetti et al., 2019b). A further 4% of patients have non-recurrent fusions engaging hematopoietic transcription factors and epigenetic regulators, e.g., *MN1* (meningioma [disrupted in balanced translocation] 1)::*FLI1* (friend leukemia integration 1 transcription factor), *BCR* (breakpoint cluster region protein):*ABL1* (ABL1, tyrosine-protein kinase ABL1), and *MAP2K2* (mitogen-activated protein kinase 2)::*MLLT10* (myeloid/lymphoid or mixed-lineage leukemia translocated to 10) (de Rooij et al., 2017). Molecular alterations are crucial for risk stratification and tailored treatment. Subgroups carrying *CBFA2T3::GLIS2*, *KMT2A*r, and *NUP98::KDM5A* are associated with poor prognosis and a high risk of disease recurrence (Figure 1B). Thus, intensive chemotherapy and hematopoietic stem cell transplantation (HSCT) are often used in first remission for these patients (de Rooij et al., 2016; de Rooij et al., 2017; Masetti et al., 2019b). In contrast, cases with *RBM15::MKL1*, *HOX*r, non-recurrent fusions, and unknown drivers have an intermediate prognosis and are treated with standard chemotherapy (Figure 1B). This review compiled molecular changes reported in pediatric non-DS AMKL (Tables 1, 2). Ongoing molecular characterization of AMKL is needed to guide future mechanistic studies into disease pathogenesis and provide essential clues on developing new targeted therapies.

**TABLE 1:** Key features of distinct molecular subgroups of pediatric non-DS AMKL.

Molecular category/characteristics	*CBFA2T3::GLIS2*	*KMT2A*r	*NUP98::KDM5A*	*RBM15::MKL1*	*HOX*r	Other (non-recurrent fusions and unknown)	DS-like AMKL (*GATA1* mutated)
Median age at diagnosis, years (range) (n)	1.2 (0.5–2.8) (n = 16) (de Rooij et al., 2017)	1.8 (0.7–7.5) (n = 15) (de Rooij et al., 2017)	1.8 (1.1–8.5) (n = 10) (de Rooij et al. (2017)	0.3 (0.1–1.4) (n = 9) (de Rooij et al., 2017)	1.5 (0.6–2.1) (n = 13) (de Rooij et al., 2017)	1.4 (0.1–12.2) (n = 16) (de Rooij et al., 2017)	1.6 (0.7–2.4) (n = 8) de Rooij et al. (2017)
	1.5 (0.5–4.0) (n = 24) (de Rooij et al., 2016)	1.9 (0.7–12.0) (n = 14) (de Rooij et al., 2016)	1.9 (0.8–8.5) (n = 14) (de Rooij et al., 2016)	0.7 (0.1–2.7) (n = 18) (de Rooij et al., 2016)		1.6 (0.1–15.1) (n = 74) (de Rooij et al., 2016)	
	0 (0–2) (n = 12) (Hara et al., 2017)						
	1.7 (1.4–1.9) (n = 4) (Lalonde et al., 2021)						
Male, % (n)	50 (n = 16) (de Rooij et al., 2017)	60 (n = 15) (de Rooij et al., 2017)	70 (n = 10) (de Rooij et al., 2017)	22 (n = 9) (de Rooij et al., 2017)	38 (n = 13) (de Rooij et al., 2017)	42 (n = 16) (de Rooij et al., 2017)	100 (n = 8) (de Rooij et al., 2017)
	325 (n = 24) (de Rooij et al., 2016)	71 (n = 14) (de Rooij et al., 2016)	343 (n = 14) (de Rooij et al., 2016)	33 (n = 18) (de Rooij et al., 2016)		51 (n = 74) (de Rooij et al., 2016)	
	50 (n = 4) (Lalonde et al., 2021)						
Median white cell count, ×10^9^/L (range) (n)	17.3 (7.5–300.1) (n = 24) (de Rooij et al., 2016)	7.4 (1.1–31.0) (n = 14) (de Rooij et al., 2016)	14.0 (5.8–188.0) (n = 14) (de Rooij et al., 2016)	13.8 (5.6–32.7) (n = 18) (de Rooij et al., 2016)		14.4 (1.1–378.5) (n = 74) (de Rooij et al., 2016)	
	33.3 (7.3–75.3) (n = 12) (Hara et al., 2017)	6.0 (4.3–24.1) (n = 3) (Hara et al., 2017)	11.8 (7.0–23.4) (n = 4) (Hara et al., 2017)	27.1 (12–42.2) (n = 2) (Hara et al., 2017)			
Additional chromosomal abnormalities (%) (n)	+21 (31%), +14 (13%), complex (25%) (n = 16 for all) (de Rooij et al., 2017)	Complex (53%), +21 (33%), +19 (27%), +6 (27%), +22 (13%) (n = 15 for all) (de Rooij et al., 2017)	Complex (90%), del (13q) (40%), +21 (20%) (n = 10 for all) (de Rooij et al., 2017)		Complex (85%), +21 (31%), +19 (31%), +8 (31%), −7 (15%) (n = 13 for all) (de Rooij et al., 2017)	Complex (44%), +21 (25%), +2 (13%), +8 (13%), −7 (13%) (n = 16 for all) (de Rooij et al., 2017)	+21 (75%), complex (50%), +19 (25%) (n = 8 for all) (de Rooij et al., 2017)
	Hyperdiploidy (58%), +21 (50%), +3 (17%), +Y (17%), complex (17%) (n = 12 for all) (Hara et al., 2017)		Complex (75%), del (13q) (75%) (n = 4 for all) (Hara et al., 2017)				
Associated mutations (%) (n)	FLT3-ITD (17%), GATA1 (17%) (n = 12 for both) (Hara et al., 2017)	NRAS (20%), JAK2 (20%), PIK3C2A (20%) (n = 15 for all) (de Rooij et al., 2017)	RB1 (90%), GATA1 (20%), PIK3CA (20%) (n = 10 for all) (de Rooij et al., 2017)		MPL (38%), JAK2 (15%), CTCF (23%), STAG2 (15%), KRAS (23%), RB1 (15%) (n = 13 for all) (de Rooij et al., 2017)	CTCF (19%), STAG2 (13%), SMC1A (13%), SMC3 (13%), MPL (13%), JAK3 (13%), PTPN11 (13%) (n = 16 for all) (de Rooij et al., 2017)	MPL (38%), JAK1 (25%), CTCF (25%), RAD21 (25%), BCOR (25%) (n = 8 for all) (de Rooij et al., 2017)
5-year EFS rate, % (n)	8 ± 7 (n = 16) (de Rooij et al., 2017)	27 ± 11 (n = 15) (de Rooij et al., 2017)	25 ± 15 (n = 10) (de Rooij et al., 2017)	53 ± 17 (n = 9) (de Rooij et al., 2017)	77 ± 12 (n = 13) (de Rooij et al., 2017)	67 ± 12 (n = 16) (de Rooij et al., 2017)	100 ± 0 (n = 8) (de Rooij et al., 2017)
5-year OS rate, % (n)	14 ± 9 (n = 16) (de Rooij et al., 2017)	27 ± 11 (n = 15) (de Rooij et al., 2017)	35 ± 16 (n = 10) (de Rooij et al., 2017)	65 ± 17 (n = 9) (de Rooij et al., 2017)	77 ± 12 (n = 13) (de Rooij et al., 2017)	80 ± 11 (n = 16) (de Rooij et al., 2017)	100 ± 0 (n = 8) (de Rooij et al., 2017)
	25 (n = 17) (Smith et al., 2020)						

n, number of patients in different studies; EFS, event-free survival; OS, overall survival. Empty cells mean there is not enough data to conclude.

**TABLE 2:** Gene signatures associated with distinct molecular subgroups of pediatric non-DS AMKL.

Molecular subgroup	Upregulated molecules or pathways	Downregulated molecules or pathways
*CBFA2T3::GLIS2*	pathways: Hippo/TNF, TGFβ/BMP, Hedgehog, ECM-receptor interaction, Super Enhancers (Smith et al., 2020; Benbarche et al., 2022)	
	molecules: cell-adhesion and cell-surface markers (e.g., CD56, CD44, ITGA2); RTKs (e.g., ROR1, MET, NTRK1); TAM family kinases (e.g., TYRO3, AXL); others (e.g., GLIS2, BMP2, HHIP, PTCH1, DHH, GLI1, RAB23, WNT9A, WNT11, ERG, DNMT3B, SLITRK5, KIT, PDGFRA, FOLR1) (Smith et al., 2020; Benbarche et al., 2022; Le et al., 2022)	molecules: CD38, CD45, HLA-DRA, HLA-DRB1, HLA-DRB5, HLA-DRB6, CASP1, TRAIL-R2, GLIPR1, PER2, GATA1 (Smith et al., 2020)
	miRNAs: miR-203a-3p, miR-452-3p, miR-452-5p, miR-135a-3p, miR-5683, miR-224-5p, miR-224-3p, miR-6507-5p, miR-130a-3p, miR-181b-5p (Smith et al., 2020)	miRNAs: miR-6503-5p, miR-196b-3p, miR-196b-5p, miR-133a-3p, and miR-5584-5p (Smith et al., 2020)
*KMT2A*r	pathway: CDK6 (Placke et al., 2014; Schmoellerl et al., 2020)	
	molecules: HOX cluster and others, such as HOXA9, MEIS1, S100A9, S100A8 (de Rooij et al., 2017; Milan et al., 2022)	molecules: associated with stem cell phenotype, including CD34, GPR56, MN1 (de Rooij et al., 2017) (Milan et al., 2022)
*NUP98::KDM5A*	pathways: JAK-STAT, CDK6 (Cardin et al., 2019; Schmoellerl et al., 2020)	pathways: Myc (Noort et al., 2021)
	molecules: HOXA cluster, HOXB cluster, MEIS1, MEIS2, E2F targets, FLT3 targets, STAT5 targets, NF1 targets, NOTCH1 targets, NEO1, MPIG6B, SELP (de Rooij et al., 2017; Cardin et al., 2019; Noort et al., 2021)	molecules: RB1 (compared to all AMKL), TP53 targets, HDAC targets (de Rooij et al., 2017; Noort et al., 2021)
*RBM15::MKL1*	pathways: Notch (Ayllon et al., 2017)	
	molecules: CDH2, ITGB1 (Ayllon et al., 2017)	
*HOX*r	molecules: HOX cluster involved in the fusion and adjacent HOX genes, such as HOXA9 and HOXA9 targets (e.g., ALDH1A1, VIM, MAFG, MAN2A2, PIM1, ADD3) (de Rooij et al., 2017)	
DS-like AMKL (*GATA1* mutated)	molecules: chromosome 21 genes (de Rooij et al., 2017)	

Empty cells mean there is not enough data to conclude.

### 2.1 *CBFA2T3::GLIS2*

The inversion inv(16)(p13.3q24.3) (generating the *CBFA2T3::GLIS2* fusion) is the most frequently, but not exclusively detected in 18%–27% of pediatric non-DS AMKL (Figure 1) (de Rooij et al., 2016; de Rooij et al., 2017; Hara et al., 2017). A newer study suggested that this lesion may be even more common, as it was identified in 4 of 6 children with non-DS AMKL (Lalonde et al., 2021). *CBFA2T3::GLIS2* is not specific to AMKL and occurs in diverse morphologic subtypes of *de novo* AML (except for acute promyelocytic leukemia (APL) and leukemia with erythroid differentiation), overall found in approximately 8.4% of cytogenetically normal AML cases (Masetti et al., 2013a; Smith et al., 2020). The inv(16)(p13.3q24.3) fuses the transcriptional repressor CBFA2T3 with the zinc finger DNA-binding transcription factor GLIS2. The prevalence of *CBFA2T3::GLIS2* appears higher in African American children, accounting for about 29% of patients with this fusion in the AAML0531 and AAML1031 cohorts (Smith et al., 2020). *CBFA2T3::GLIS2* characterizes an extremely aggressive leukemic subgroup with a grim prognosis across all differentiation subtypes (overall survival rates 14%–40%) (Gruber and Downing, 2015; de Rooij et al., 2016; de Rooij et al., 2017; Smith et al., 2020). Among *CBFA2T3::GLIS2* AMKL, approximately 40% of patients also carry a *DHH* (desert hedgehog):*RHEBL1* (Ras homologue enriched in brain like 1) fusion (Masetti et al., 2013b; Jetten, 2019). The co-occurrence of these two fusions has an even poorer prognosis than the *CBFA2T3::GLIS2* alone (Masetti et al., 2013b; Jetten, 2019). Children with *CBFA2T3::GLIS2* AML (including AMKL) tend to be younger (median age of onset ∼1.5 years) and display distinct clinical and laboratory features, including stronger expression of CD56 (neural cell adhesion molecule, NCAM) and a more frequent extramedullary involvement compared to other AML (Masetti et al., 2013a; de Rooij et al., 2016; Hara et al., 2017; Smith et al., 2020; Zangrando et al., 2021). Standard karyotyping cannot identify the *CBFA2T3::GLIS2*, because of its cryptic nature. Immunophenotypic features may suggest the presence of this fusion, as blasts carrying *CBFA2T3::GLIS2* have bright CD56 expression with dim-to-negative HLA-DR (human leukocyte antigen DR isotype), CD38, and CD45 referred to as the RAM immunophenotype (Zangrando et al., 2021). The somatic mutational burden of *CBFA2T3::GLIS2* subtype is much lower than in other AMKL subgroups (Gruber et al., 2012; Dang et al., 2017; Masetti et al., 2019a). However, mutations in tyrosine kinases such as FLT3 (fms-like tyrosine kinase 3), KIT (tyrosine-protein kinase KIT), RAS (Rat sarcoma), JAK/STAT (Janus kinases/signal transducer and activator of transcription), and transcription factor GATA1 can co-occur (Gruber et al., 2012; de Rooij et al., 2017; Hara et al., 2017). *CBFA2T3::GLIS2* can also be accompanied by trisomies of chromosomes 3, 21 and 8, complex karyotype, or hyperdiploidy (Hara et al., 2017; Amano et al., 2020; Smith et al., 2020; Gillam et al., 2022).

**FIGURE 2:**
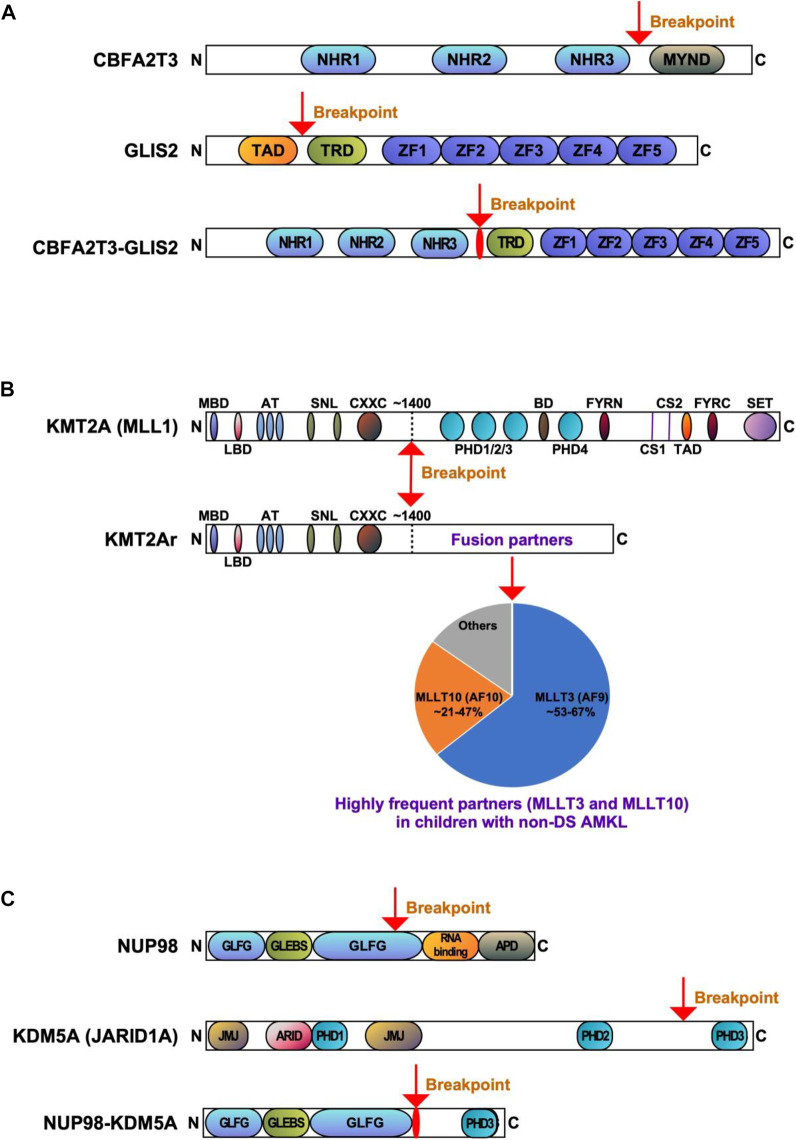
Recurrent gene fusions associated with poor outcomes in pediatric non-DS AMKL. Schematics display the functional domains but are not drawn to scale. The breakpoints are indicated by arrows. **(A)**. Structure of CBFA2T3, GLIS2, and CBFA2T3-GLIS2 proteins. NHR, nervy homology region; MYND, myeloid, nervy, and DEAF-1; TAD, transactivation domain; TRD, transcriptional repression domain; ZF, zinc finger. **(B)** Structure of KMT2A (MLL) and KMT2Ar (chimeric) proteins. MBD, menin-binding domain; LBD, LEDGF-binding domain; AT, AT hooks; SNL, speckled nuclear localization domains; CXXC, CXXC domain; PHD1/2/3/4, plant homeodomain 1/2/3/4; BD, bromodomain. CS1 and CS2 are the taspase-1 cleavage sites, and FYRN and FYRC are the domains whereby KMT2A-N and KMT2A-C interact after cleavage. TAD, transactivation domain; SET, H3K4 histone methyltransferase domain. KMT2A fusion proteins are caused by chromosomal rearrangements leading to an in-frame fusion between the N-terminus of KMT2A and one of the multiple fusion partners. The breakpoints of KMT2A are located in the region of ∼1400 aa that contains the AT-hook DNA binding motifs and CXXC domains binding unmethylated CpG-containing DNA. Frequencies of the KMT2A fusion partner proteins are shown, with transcription cofactors MLLT3 (AF9) (found in 53%–67% of cases) and MLLT10 (AF10) (found in 21%–47% of cases) being the most common (de Rooij et al., 2016; de Rooij et al., 2017; Hara et al., 2017). **(C)** Structure of NUP98, KDM5A, and NUP98-KDM5A proteins. GLFG, GLFG repeats; GLEBS, Gle2-binding domain; RNA binding, RNA binding domain; APD, autoproteolytic domain; JMJ, Jumonji domain; ARID, AT-rich interactive domain; PHD1/2/3, plant homeodomains 1/2/3.

*CBFA2T3::GLIS2* fuses the 5′ portion of *CBFA2T3* (also named as *ETO2*, *MTG16*, *RUNX1T3* or *ZMYND4*) in frame with the 3’ region of *GLIS2*. The majority of chimeric *CBFA2T3::GLIS2* fusions (80%) are between exon 11 of *CBFA2T3* and exon 3 of *GLIS2*. Other rare fusion transcripts have also been reported, including between exons 9, 10 or 12 of *CBFA2T3* and exons 2 or 3 of *GLIS2* (Masetti et al., 2019b; De Marchi et al., 2019; Amano et al., 2020; Smith et al., 2020). The chimeric protein generated by the most common fusion retains most of the functional domains of both proteins with the loss of myeloid, nervy and DEAF-1 domain (MYND) that interacts with nuclear receptor corepressor complex in CBFA2T3, as well as the loss of transactivation domain (TAD) that mediates transcriptional activation of target genes in GLIS2 (Figure 2A). Both CBFA2T3 and GLIS2 participate in leukemic transformation driven by CBFA2T3::GLIS2 (Thirant et al., 2017a).

CBFA2T3 is a member of the RUNX1T1 (runt-related transcription factor 1, translocated) complex. CBFA2T3 belongs to the eight-twenty-one (ETO) family of chromatin-associated proteins, functioning as a master transcriptional coregulator and a Wnt (wingless/integrated) and Notch (neurogenic locus notch homolog protein) signaling suppressor in normal and malignant hematopoiesis (Steinauer et al., 2017; Masetti et al., 2019a; Steinauer et al., 2019; Steinauer et al., 2020; Jakobczyk et al., 2021). CBFA2T3 is expressed in all hematopoietic cells and contains three Nervy homology region (NHR) domains and a MYND domain (Figure 2A). CBFA2T3 participates in hemopoietic stem cell (HSC) self-renewal and differentiation, megakaryocyte-erythrocyte progenitor development, and leukemia stem cell (LSC) expansion (Steinauer et al., 2017; Masetti et al., 2019a; Steinauer et al., 2019; Steinauer et al., 2020; Jakobczyk et al., 2021). Physiological binding partners for CBFA2T3 include transcription factors and chromatin modifiers, and it is generally presumed that CBFA2T3 represses gene transcription through binding to multiple corepressors, such as E-proteins (e.g., E2A basic helix-loop-helix transcription factor), nuclear receptor corepressors (NCOR), and histone deacetylases (HDAC) (Steinauer et al., 2017). CBFA2T3 contributes to transcriptional repression via GATA1-SCL (stem cell leukemia, also known as TAL1) complex during megakaryopoiesis (Schuh et al., 2005; Hamlett et al., 2008). In the megakaryoblastic cell line L8057, *CBFA2T3* knockdown enhances megakaryocytic differentiation via elevating gene expression associated with terminal megakaryocytic maturation (Hamlett et al., 2008); whilst *CBFA2T3* deletion in a mouse model induces differentiation along the granulocytic-monocytic lineages at the expense of erythroid-megakaryocytic differentiation (Chyla et al., 2008).

GLIS2 is a member of the GLIS subfamily of Krüppel-like zinc finger transcription factors closely related to the GLI and ZIC subfamilies (Scoville et al., 2017). GLIS2 contains a TAD, a transcriptional repression domain (TRD), and a DNA binding domain (five zinc finger motifs) (Figure 2A). GLIS2 function is implicated in the processes of hematopoiesis and leukemogenesis (Jetten, 2019; Pinto and Chetty, 2020). Normally, GLIS2 is not expressed in the differentiating hematopoietic cells (Gruber et al., 2012; Thirant et al., 2017b); however, its expression contributes to HSC repopulation (Holmfeldt et al., 2016). Knockdown of *GLIS2* in murine LSK (Lin-SCA1+c-KIT+) cells reduces HSC repopulation, suggesting GLIS2 could regulate HSC engraftment and hematopoietic reconstitution (Holmfeldt et al., 2016). *GLIS2* overexpression in *MOZ* (monocytic leukemia zinc finger protein):*TIF2* (transcriptional intermediary factor 2) leukemic cells promotes their differentiation into mature myeloid cells and delays AML development in mice, indicating that GLIS2 inhibits AML initiation by inducing LSC differentiation (Shima et al., 2018).

Although the molecular bases for AMKL transformation by *CBFA2T3::GLIS2* are still far from being elucidated, distinct functional properties of this chimeric protein have been illustrated (Masetti et al., 2019a). *CBFA2T3::GLIS2* AMKL has a unique gene expression pattern (Gruber et al., 2012; Thirant et al., 2017a; Benbarche et al., 2022). *GATA1* downregulation, *ERG* (ETS-related gene) upregulation, and activation of super enhancer elements may impair megakaryocytic differentiation and increase abnormal self-renewal of leukemic cells in AMKL associated with *CBFA2T3::GLIS2* (Thirant et al., 2017a; Benbarche et al., 2022). Super enhancers are clusters of regulatory elements characterized by high intensity of enhancer-related histone tail modifications (Benbarche et al., 2022). Ectopic expression of *CBFA2T3::GLIS2* induces the formation of super enhancers, which controls the expression of KIT and platelet-derived growth factor receptor alpha (PDGFRA) involved in leukemic progression (Benbarche et al., 2022). *CBFA2T3::GLIS2* positive cases also display higher expression of CD56, a CD56-interacting partner CACNB2 (calcium voltage-gated channel auxiliary subunit beta 2), GABRE (gamma-aminobutyric acid type A receptor subunit epsilon), miR-224 and miR-452 (Smith et al., 2020). Both miR-224 and miR-452 are intronic miRNAs transcribed from the *GABRE* locus and have numerous mRNA targets involved in immune responses, leukocyte activation, and leukocyte differentiation. Upregulation of other miRNAs (e.g., miR-181b-5p) may be responsible for the reduction in expression of apoptotic and tumor suppressor genes, and simultaneous downregulation of tumor suppressive miRNAs (e.g., miR-196a/b, miR-133a, and miR-199a/b) (Smith et al., 2020). The role of miRNA changes in leukemia initiation by *CBFA2T3::GLIS2* is not well defined. Overexpression of miR-181b-5p downregulates apoptotic genes *CASP1* (caspase 1) and *TRAIL-R2* (TNF-related apoptosis-inducing ligand receptor 2), and the circadian rhythm gene *PER2* (period circadian regulator 2), while loss of miR-196b is associated with *ITGA2* (integrin subunit alpha 2) and *DNMT3B* (DNA methyltransferase 3 beta) overexpression (Smith et al., 2020). When highly expressed, *ITGA2* and *DNMT3B* are independent indicators of poor prognosis in AML (Lamba et al., 2018; Lian et al., 2018). *CBFA2T3::GLIS2* is also associated with an overexpression of several cell adhesion and cell surface molecules (e.g., extracellular matrix binding, cell adhesion, and integrin binding genes) and signaling pathways (e.g., Hippo, transforming growth factor beta (TGFβ), bone morphogenetic proteins (BMP), JAK/STAT and Hedgehog) (Gruber et al., 2012; Smith et al., 2020). Overall, multiple molecular alterations may contribute to the phenotype of *CBFA2T3::GLIS2* leukemia.

Considering the lack of other recurrent mutations in patients carrying *CBFA2T3::GLIS2*, this fusion probably is the primary genomic alteration. However, the evidence is mixed on whether *CBFA2T3::GLIS2* alone is sufficient for leukemic transformation. On the one hand, the introduction of *CBFA2T3::GLIS2* into murine bone marrow is insufficient to induce overt leukemia in mice (Dang et al., 2017). On the other hand, the transduction of human CD34^+^ cord blood stem cells with a lentivirus carrying *CBFA2T3::GLIS2* leads to increased proliferation, maturation arrest, and morphologic and immunophenotypic aberrations consistent with AMKL (Le et al., 2022). Further, an inducible transgenic mouse model demonstrated that *CBFA2T3::GLIS2* expressed in fetal HSCs leads to a rapid and aggressive AMKL, whereas its expression in adult bone marrow HSCs results in AML (Lopez et al., 2019). In an endothelial cell coculture system, the expression of *CBFA2T3::GLIS2* transforms human cord blood hematopoietic stem and progenitor cells (HSPCs) and leads to highly aggressive leukemia in mice (Le et al., 2022).

In summary, cytogenetically cryptic *CBFA2T3::GLIS2* fusion is a potent oncogene contributing to malignant transformation and an extremely lethal and treatment-refractory AML. Disease expressing this fusion have unique molecular features with a specific gene expression signature, dysregulated expression of cell adhesion molecules, RTKs, and signaling pathways (e.g., Hippo, TGFβ, BMP, JAK/STAT and Hedgehog). Despite the use of intensive chemotherapy and HSCT, the outcomes of *CBFA2T3::GLIS2* leukemia are poor. The mechanism of leukemogenesis associated with *CBFA2T3::GLIS2* remains incompletely understood. Therefore, further mechanistic studies are required to inform the development of novel therapies.

### 2.2 *KMT2A* rearrangements

Alterations of the *KMT2A* gene (also known as mixed lineage leukemia 1, *MLL1* or *MLL* gene) located at 11q23.3 are found in both acute lymphoblastic leukemia (ALL) and AML, making up ∼10% of all leukemia cases in all age groups (Winters and Bernt, 2017). *KMT2A* rearrangements (*KMT2A*r) are one of the most common recurrent genetic aberrations found in 15%–25% of all newly diagnosed cases of pediatric AML (Meyer et al., 2006; Meyer et al., 2009; Meyer et al., 2013; Meyer et al., 2018; Hoffmeister et al., 2021; Quessada et al., 2021; Meyer et al., 2023; Yuen et al., 2023). *KMT2A*r are particularly prevalent in infant AML, accounting for approximately 30% of children presenting below the age of 2 years. *KMT2A*r correlate with monoblastic and monocytic AML subtypes (71.5%–73%) and are much less common in other subtypes (Meyer et al., 2023). In pediatric AMKL, *KMT2A*r are reported in 7%–17.4% of patients, with the median age at diagnosis of 1.9 years (range 0.7–12 years) (Chen and Armstrong, 2015; de Rooij et al., 2016; de Rooij et al., 2017; Hara et al., 2017; Masetti et al., 2019b; Maarouf et al., 2019). Numerous *KMT2A* fusion partners have been identified in children with AMKL, including *MLLT1* (myeloid/lymphoid or mixed-lineage leukemia translocated to 1, also known as *ENL*), *AFF1* (AF4/FMR2 family member 1, previously known as *AF4*), *MLLT3* (also known as AF9), *MLLT6*, *MLLT9*, *MLLT10* (also known as *AF10*), and *SEPT9* (septin 9) (Takita et al., 2009; de Rooij et al., 2016; de Rooij et al., 2017; Hara et al., 2017; Forlenza et al., 2018; Lalonde et al., 2021; Qiu et al., 2022). The two most common *KMT2A* translocations in AMKL are t(9;11)(p21;q23) (generating *KMT2A::MLLT3* fusion) and t(10;11)(q12;q23) (generating *KMT2A::MLLT10* fusion) (Figure 2B). Translocation t(9;11)(p21;q23) was found in 8 of 15 (53%), 9 of 14 (64%) and 2 of 3 (66.7%) of patients in three different studies (de Rooij et al., 2016; de Rooij et al., 2017; Hara et al., 2017). In the same studies, t(10;11)(q12;q23) was the second most common, detected in 7 of 15 (46.7%), 3 of 14 (21.4%), and 1 of 3 (33.3%) of patients, respectively (de Rooij et al., 2016; de Rooij et al., 2017; Hara et al., 2017). The prognosis of *KMT2A*r AML varies, heavily relying on its translocation partner. However, in pediatric AMKL *KMT2A*r are always regarded as high-risk fusion events linked to a greater risk of relapse and poorer overall survival (de Rooij et al., 2016; de Rooij et al., 2017). *KMT2A*r subgroup of pediatric non-DS AMKL tends to have higher white cell count (WCC) and is more common in males, although these differences were not statistically significant in the study by de Rooij et al. (de Rooij et al. 2016). Pediatric *KMT2A*r AML was shown to have a higher expression of CD33 compared to *KMT2A* wild-type AML (Pollard et al., 2021). However, it is unclear whether pediatric *KMT2A*r AMKL has the same pattern, so further studies are required. Co-occurring mutations can be found in AMKL associated with *KMT2A*r, including in molecules such as NRAS (neuroblastoma RAS viral oncogene homolog), KRAS (Ki-ras2 Kirsten rat sarcoma viral oncogene homolog), PTPN11 (tyrosine-protein phosphatase non-receptor type 11), NF1 (neurofibromatosis type 1), EPOR (erythropoietin receptor), MPL (thrombopoietin receptor), JAK1/2/3, PIK3C2A (phosphatidylinositol-4-phosphate 3-kinase catalytic subunit type 2 alpha), KIT, cohesin and epigenetic regulators, e.g., STAG3 (stromal antigen 3), SETD2 (SET domain containing 2, histone lysine methyltransferase), IDH1 (isocitrate dehydrogenase 1), CREBBP (cyclic adenosine monophosphate response element binding protein binding protein), transcription factors, e.g., GATA1, RNA splicing and regulatory proteins, e.g., U2AF1 (U2 small nuclear RNA auxiliary factor 1) and DDX3X (DEAD-Box helicase 3 X-linked) (de Rooij et al., 2017; Foster et al., 2019). *KMT2A*r associated with *KRAS* mutations confer a particularly poor prognosis (Matsuo et al., 2020). Several additional chromosomal abnormalities can be observed in pediatric AMKL with *KMT2A*r, such as trisomy 8, trisomy 21, hyperdiploidy, monosomy 15, and a complex karyotype (de Rooij et al., 2016; de Rooij et al., 2017; Hara et al., 2017). The clinical impact of these co-occurring chromosomal changes remains unclear.

KMT2A represents a key transcription factor and histone-H3 lysine-4 (H3K4) methyltransferase that serves as a master controller for the transcription of critical genes implicated in normal embryonic development and hematopoiesis (Li and Song, 2021; Sugeedha et al., 2021). KMT2A is a large protein (500 kDa, 3696 amino acids) and contains multiple conserved domains, including menin binding motif, LEDGF (lens epithelium-derived growth factor) binding domain, AT-hook (DNA binding) motifs, nuclear localization signals, CXXC domain (nonmethylated CpG DNA binding domain), PHD (plant homeodomain) fingers, bromodomain, FYRN (FY-rich domain N-terminal) domain, taspase 1 cleavage sites, TAD, FYRC (FY-rich domain C-terminal), and SET domains (Li and Song, 2021) (Figure 2B). Interestingly, its N-terminal ∼1400 residues, containing the AT-hook and CXXC domains, act as a transcription factor that recognizes and binds target genes such as HOXA9 and MEIS1 (Meis homeobox 1) (Muntean et al., 2010). The C-terminal SET domain functions as an H3K4 methyltransferase that mediates chromatin modifications associated with epigenetic transcriptional activation (Li and Song, 2021). An oncogenic fusion protein produced by *KMT2A*r predominantly consists of the N-terminal DNA-interacting domains of KMT2A (residues 1–∼1400) fused in frame with one of over 100 fusion partners (Li and Song, 2021) (Figure 2B). KMT2A fusions act as oncoproteins in different leukemic cell and animal models, and expression of those fusions can promote proliferation and arrest myeloid differentiation of hematopoietic progenitors, resulting in their accumulation (Slany, 2016; Skucha et al., 2018). It appears that all forms of KMT2A fusion oncoproteins positively regulate the expression of *HOX* genes and the HOX cofactor MES1 critical for the leukemic transformation of hematopoietic progenitors (Muntean et al., 2010; Chen and Armstrong, 2015). The mechanism of leukemogenesis may therefore rely on the upregulation of target genes by KMT2A fusion proteins and other recruited proteins, e.g., super elongation complexes involving DOT1L (DOT1 like histone lysine methyltransferase) and H3K79 (lysine 79 of histone H3), or polycomb repressive complex 1 (Chen and Armstrong, 2015; Slany, 2016; Li and Song, 2021). *KMT2A::MLLT3*, the most common form of *KMT2A*r in children with non-DS AMKL, is sufficient to induce a myeloproliferative disorder in mice and to generate leukemia in a mouse model using healthy CD34^+^ cord blood HSPCs (Mulloy et al., 2008; Wei et al., 2008; Stavropoulou et al., 2016). Wild-type MLLT3 is a positive regulator of early erythroid and megakaryocytic cell fates in primitive human cord blood cells, together with GATA1 (Pina et al., 2008). In a mouse model generated using human CD34^+^ cord blood HSPCs containing *KMT2A::MLLT3*, the development of a pronounced hypomethylation phenotype is an early event during leukemogenesis (Milan et al., 2022). Leukemia development in this model was associated with the loss of expression of stem-cell associated genes, gain of expression of HOXA9 and MEIS1, and increased expression ratio of S100 (S100 calcium-binding proteins) A8 to A9 proteins. KMT2A fusion proteins, particularly KMT2A::ENL, regulate only a small subset of genes recognized by wild-type KMT2A (e.g., *HOXA9*, *MEIS1*, and several other transcription factor genes, e.g., *EYA1* (eyes absent homolog 1), *SIX1* (SIX homeobox 1) and *SIX4* (SIX homeobox 4), highlighting that the transforming capacity of *KMT2A* fusion may not be limited to *HOXA*/*MEIS1* genes (Wang et al., 2011). Another study showed that IKAROS (IKAROS family zinc finger 1) acts as an essential regulator in *KMT2A*r AML by influencing tumor suppressor pathways, immune dysregulation, and changes in cell differentiation (Aubrey et al., 2022). Cyclin-dependent kinase 6 (CDK6), a cell cycle regulator, was also shown to play a role in the development of *KMT2A*r AML (Placke et al., 2014). Inhibition of CDK6 by shRNA or pharmacological inhibitor (e.g., PD-0332991) decreases leukemic cell growth and promotes myeloid cell differentiation in cell lines and primary human AML cells harboring different *KMT2A* translocations. In a mouse model with *KMT2A::MLLT3*, CDK6 inhibition increases cell differentiation and prolongs mice survival (Placke et al., 2014).

In summary, similar to *CBFA2T3::GLIS2*, *KMT2A*r in pediatric non-DS AMKL drive disease with inferior outcomes, high incidence of treatment non-response and relapse. Children carrying *KMT2A*r present with distinct clinical and molecular features, including monocytic differentiation, higher WCC, higher expression of CD33, fewer co-occurring mutations compared with *KMT2A* wild-type AML, and higher expression of *HOXA*/*MEIS1* genes. However, despite improvements in the molecular characterization of *KMT2A*r AML, leukemogenic mechanisms remain incompletely understood, and new therapeutic approaches are needed.

### 2.3 *NUP98::KDM5A* and other *NUP98* rearrangements

The cytogenetically cryptic translocation t(11;12)(p15;q35) (resulting in *NUP98::KDM5A* fusion, also known as *NUP98::JARID1A* or *NUP98:: RBP2*) was initially identified in pediatric AMKL in 2006 (van Zutven et al., 2006). *NUP98::KDM5A* fuses *NUP98* located on chromosome 11p15 with *KDM5A* located on the telomeric end of chromosome 12p13.3 (Masetti et al., 2019b). The fusion is found in 2% of pediatric AML overall, primarily in 8%–12% of non-DS AMKL and 12% of infant AML (de Rooij et al., 2013; de Rooij et al., 2016; de Rooij et al., 2017; Masetti et al., 2019b; Hara et al., 2020; Noort et al., 2021). *NUP98::KDM5A* AML patients have a median age at diagnosis of 3.2 years (ranging from 3 weeks to 18.5 years) (Noort et al., 2021). *NUP98::KDM5A* can be found in all morphologic subtypes of AML except for APL. Megakaryocytic differentiation was seen in 34% of *NUP98::KDM5A* AML, monocytic in 21%, and erythroid in 17% of these cases (Noort et al., 2021). The median age of AMKL patients carrying *NUP98::KDM5A* is lower than in AML overall (1.8–1.9 years, range: 0.7–12 years) (de Rooij et al., 2016; de Rooij et al., 2017; Noort et al., 2021). *NUP98::KDM5A* confers a dismal prognosis with an overall survival of 33%–36% due to a high incidence of induction failure or relapse (de Rooij et al., 2016; Hara et al., 2020; Noort et al., 2021). Additional chromosomal abnormalities can be found in association with *NUP98::KDM5A*, in particular trisomy 21, hyperdiploidy, −13/-13q and a complex karyotype (de Rooij et al., 2016).

*NUP98* has many different partner genes (over 30) to produce a series of abnormal fusion proteins in several hematopoietic malignancies (including AML, myelodysplastic syndrome, T-ALL, and mixed-phenotype acute leukemia) (Michmerhuizen et al., 2020). *KDM5A* is the most common fusion partner of *NUP98* in AML and AMKL (de Rooij et al., 2017; Cardin et al., 2019), and *NSD1* (nuclear receptor) is the second most common (Mercher and Schwaller, 2019). NUP98::KDM5A and NUP98::NSD1 chimeric proteins fuse the N-terminus of NUP98 with the C-terminus of KDM5A (harboring PHD3) or NSD1 (harboring the PHD and SET domains), respectively (Wang et al., 2007; Wang et al., 2009; Hollink et al., 2011; Schmoellerl et al., 2020). *NUP98::KDM5A* patients have distinct clinical features compared with *NUP98::NSD1*, presenting at a younger age and showing a lower WCC (Noort et al., 2021). Additional mutations that associate with *NUP98::NSD1*, particularly those affecting *RAS*, *WT1* and *FLT3*, are rarely found in association with *NUP98::KDM5A* (Noort et al., 2021). In contrast, loss of *RB1* (Retinoblastoma 1) locus frequently associates with *NUP98::KDM5A* (90%, n = 9), and *GATA1* mutations were present in 2 of 9 *NUP98::KDM5A* patients with the *RB1* loss (de Rooij et al., 2017; Masetti et al., 2019b; Iacobucci et al., 2019; Michmerhuizen et al., 2020). Leukemic cells carrying *NUP98::KDM5A* show a strong upregulation of *HOXA* (*HOXA5*, *HOXA9*, *HOXA10*), and *HOXB* (*HOXB2*, *HOXB3*, *HOXB4*, *HOXB5*, *HOXB6*) genes compared with *RBM::MKL1* and *CBFA2T3::GLIS2* fusions (de Rooij et al., 2013; Noort et al., 2021). Intriguingly, this upregulation of *HOX* genes is shared with *NUP98::NSD1*, *DEK* (DEK proto-oncogene)::*NUP214* (nucleoporin 214), and *NPM1* (nucleophosmin 1) mutated cases, suggesting *HOXA* and *HOXB* overexpression is a common alteration in leukemia development (de Rooij et al., 2013; Noort et al., 2021). Additionally, both *NUP98::KDM5A* and *NUP98::NSD1* AML show upregulation of targets of E2F (E2F transcription factor 1) and FLT3, and downregulation of targets of TP53 and HDAC (Noort et al., 2021). The upregulation of STAT5, NF1, and NOTCH1, and the downregulation of MYC targets were also identified in *NUP98::KDM5A* cases, but not in *NUP98::NSD1* cases (Noort et al., 2021).

*NUP98::KDM5A* is undetectable with conventional karyotyping, whereas newly developed next-generation sequencing (NGS) technologies can detect it (Mercher and Schwaller, 2019). The most common in-frame fusion is between exon 13 of *NUP98* and exon 27 of *KDM5A*, but an in-frame fusion involving exon 14 of *NUP98* has also been described (de Rooij et al., 2013). NUP98:KDM5A protein contains the N-terminal glycine-leucine-phenylalanine-glycine (GLFG) repeats of NUP98 fused to the C-terminal PHD3 finger of KDM5A (Figure 2C) (Gough et al., 2011; Cardin et al., 2019). Both, the GLFC repeat of NUP98 and the PHD3 domain of KDM5A are thought to participate in leukemic transformation (Wang et al., 2009; Gough et al., 2011; Michmerhuizen et al., 2020).

NUP98 is a structural component of a nuclear pore complex responsible for transporting small ions, polypeptides and macromolecules (e.g., RNA and proteins) into and out of the nucleus (Gough et al., 2011; Michmerhuizen et al., 2020). NUP98 also functions as a transcriptional regulator and assists cell cycle progression (Gough et al., 2011). NUP98 protein consists of GLFG repeats, GLEBS (Gle2-binding sequence) binding domain, RNA-binding sites, and autoproteolytic cleavage site (Figure 2C). GLFG repeats are thought to function as docking sites for karyopherins (that support molecular trafficking), CREBBP, p300 (EP300), exportin 1 (XPO1), and the mRNA export factor TAP (Gough et al., 2011; Michmerhuizen et al., 2020). In hematopoietic progenitors, NUP98 regulates H3K4me3 (trimethylation of histone H3 at lysine 4) via binding to promoters adjacent to regions associated with H3K4me3 and via interaction with Wdr82-Set1A/COMPASS (complex of proteins associated with Set1) (Franks et al., 2017). The role of NUP98 in leukemia development depends on its interaction with the PHD3 domain in KDM5A (Wang et al., 2009; Cardin et al., 2019; Zhang et al., 2020).

KDM5A is a histone lysine demethylase that can remove methyl groups from histones H3K4me1/2/3, thus modulating transcriptional activation or repression (Yang et al., 2021). KDM5A is composed of a Jumonji (JMJ) N domain, a Bright/ARID DNA binding domain, a JMJ C domain, and three PHD domains (Figure 2C) (Yang et al., 2021). PHD3 finger is capable of binding to H3K4me1/2/3, with H3K4me3 being the preferred substrate (Yang et al., 2021). *KDM5A* overexpression in leukemia associates with a poor prognosis (Gale et al., 2016; Yang et al., 2021).

*NUP98::KDM5A* fusion is sufficient to induce leukemic transformation by altering proliferation, differentiation, maturation, and self-renewal in different cellular and animal models (Wang et al., 2009; Cardin et al., 2019; Domingo-Reines et al., 2022). The bone marrow-derived HSPCs transduced with *NUP98::KDM5A* fusion show myeloid differentiation arrest and sustained self-renewal (Wang et al., 2009). Overexpression of *NUP98::KDM5A* in human cord blood stem/progenitor cells results in maturation block and abnormal proliferation with short latency (Cardin et al., 2019). Similarly, in a human embryonic stem cell (hESC) model with doxycycline-regulated *NUP98::KDM5A* expression, inducible expression of the fusion protein affects progenitor cell production, accompanied by enhanced expression of *HOXA* gene cluster (Domingo-Reines et al., 2022). Mice transplanted with *NUP98::KDM5A* bone marrow progenitors develop CD34^+^CD117^+^ AML, characterized by transcriptional upregulation of lineage-specific factors (HOXA, GATA3, MEIS1, EYA, PBX1 (pre-B-cell leukemia transcription factor 1), and epigenetic activation of the *HOXA* gene cluster (Wang et al., 2009). Transfer of human cord blood HSPCs modified to express *NUP98::KDM5A* in mice results in AML, including AMKL (Cardin et al., 2019). Expression profiling of these synthetic AMKL xenografts closely matches those of AMKL patients (correlation coefficients >0.9). Prominent expression changes include transcriptional upregulation of *HOXA*, *HOXB*, *MEIS1* and *MEIS2*, epigenetic activation of *HOXB*, and overactivation of STAT5A signaling (Cardin et al., 2019). Integrative analysis of transcriptomic and proteomic data from *NUP98::KDM5A* AMKL models identified cell membrane proteins SELP (selectin P), MPIG6B (megakaryocyte and platelet inhibitory receptor G6b), and NEO1 (neogenin) as novel disease biomarkers (Cardin et al., 2019). Upregulation of JAK-STAT signaling occurs in both synthetic AMKL xenografts and *NUP98*-rearranged AMKL patient-derived xenografts (Cardin et al., 2019). Like in *KMT2A*r AML, CDK6 is highly expressed in *NUP98* rearranged AML, representing a critical direct target of NUP98 fusion proteins (Schmoellerl et al., 2020). CDK6 expression is essential for initiating and maintaining AML driven by *NUP98* fusion. CDK6 inhibitor palbociclib induces myeloid differentiation, apoptosis and cell cycle arrest *in vitro* and *in vivo* (Schmoellerl et al., 2020). Thus, CDK6 inhibition has been proposed as a rational strategy to target *NUP98* fusion effects in AML (Schmoellerl et al., 2020).

Although the detailed molecular mechanism of leukemic transformation by *NUP98::KDM5A* is unclear, it appears that the upregulation of *HOX*, *GATA3*, *MESI1* and *PBX1* transcription factors are essential (Wang et al., 2009; Franks et al., 2017). The N-terminal domain is responsible for recruiting the WDR82-SET1A/B-COMPASS complex to promote H3K4me through which it upregulates gene expression (Franks et al., 2017). The C-terminal PHD3 domain that recognizes H3K4me3 also contributes (Wang et al., 2009). Cell transformation is dependent on the integrity of the PHD3 finger that specifically binds H3K4me2/3. Mutations affecting conserved residues in this domain abrogate H3K4me3 binding, which prevents the binding of NUP98:KDM5A protein to the *HOXA9* promoter (Wang et al., 2009).

In summary, *NUP98::KDM5A* fusion identifies a high-risk group of patients with inferior outcomes. Leukemic cells harboring *NUP98::KDM5A* have unique molecular features. NUP98:KDM5A is recruited to the promoters of *HOX* genes associated with H3K4me3/2. This causes transcriptional upregulation of *HOXA*, *GATA3*, *MEIS1*, *MEIS2*, *EYA*, and *PBX1*, and epigenetic activation of the *HOXA* and *HOXB* gene clusters. Upregulation of these genes blocks differentiation and maturation, and sustains proliferation and self-renewal of leukemic cells. Nevertheless, the exact molecular mechanism of malignant transformation driven by *NUP98::KDM5A* is not fully understood. The pathogenetic significance of the *RB1* loss in the presence of *NUP98::KDM5A* is unknown. Novel molecularly guided treatment options are required for this disease.

### 2.4 *RBM15::MKL1*

**FIGURE 3:**
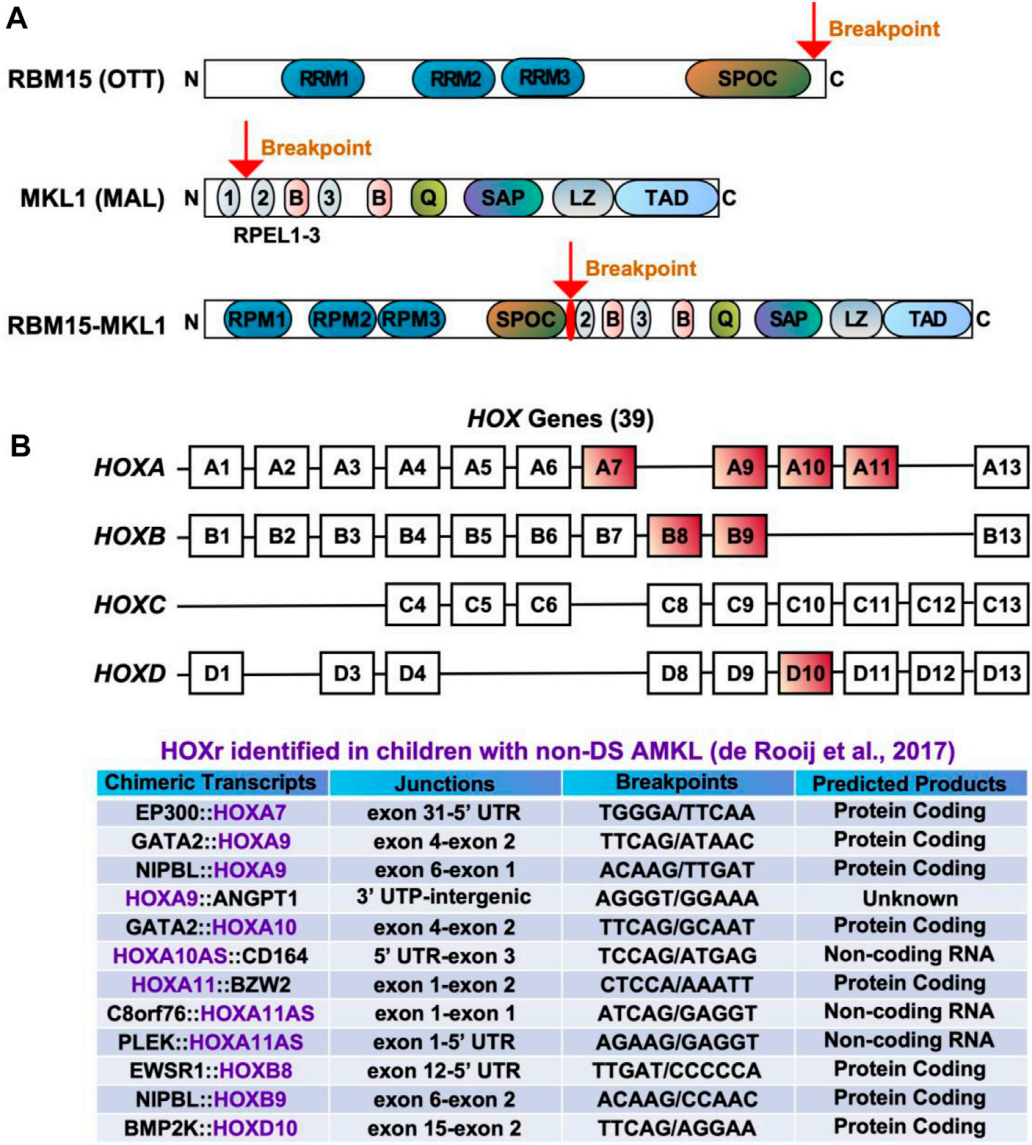
Chimeric fusions associated with favorable outcomes in pediatric non-DS AMKL. **(A)** Structure of RBM15 (OTT), MKL1 (MAL), and RBM15-MKL1 proteins. RRM, RNA recognition motif; SPOC, Spen paralogue and orthologue C-terminal domain; RPEL, actin-binding motifs with Arg-Pro-X-X-X- Glu-Leu core consensus; B, basic domains; Q, glutamine-rich domain; SAP, homology domain found in SAF-A/B, acinus, PIAS; LZ, leucine-zipper-like domain; TAD, transactivation domain. **(B)** *HOX* genes and *HOX*r identified in pediatric non-DS AMKL (de Rooij et al., 2017). There are 39 *HOX* genes clustered into four families: *HOXA*, *HOXB*, *HOXC* and *HOXD*. *HOXA7*, *HOXA9*, *HOXA10*, *HOXA11*, *HOXB8*, *HOXB9* and *HOXD10* are rearranged in AMKL, with *HOXA9*, *HOXA10* and *HOXA11* being the most frequently affected (de Rooij et al., 2017).

The t(1;22)(p13;q13) results in a fusion of the *RBM15* (the RNA-binding motif protein 15) gene, also named as *OTT* (one twenty-two) located on chromosome 1p13 to the *MKL1* (megakaryoblastic leukemia-1) gene, also named as *MAL* (megakaryocytic acute leukemia) or *MRTFA* (myocardin-related transcription factor A) located on chromosome 22q13 (Ma et al., 2001; Raffel et al., 2007). Although the translocation t(1;22)(p13;q13) is rare in pediatric AML (seen in approximately 0.3% of cases) (Quessada et al., 2021), it is almost exclusively seen in infants or young children (age <3 years) with AMKL (10%–15% of pediatric non-DS AMKL) (Carroll et al., 1991; de Rooij et al., 2016; Masetti et al., 2019b; Quessada et al., 2021). The t(1;22)(p13;q13) AMKL is associated with young patient age (median 5–8 months), female prevalence, and intermediate risk (Inaba et al., 2015; de Rooij et al., 2016; Masetti et al., 2019b). Because of the high selectivity of the t(1;22)(p13;q13) for infants with AMKL, the hypothesis was raised that this translocation may arise *in utero*, causing transformation of a unique developmental stage in HSPCs (Reed et al., 2021). Children with t(1;22)(p13;q13) younger than 6 months do not harbor other cytogenetic abnormalities, while children older than 6 months may have hyperdiploidy with the duplication of der(1)t(1;22) or gains of chromosomes 2, 6, 19, or 21 (de Rooij et al., 2016; Quessada et al., 2021). The RBM15::MKL1 chimeric protein contains all known functional domains of RBM15 and MKL1 (Figure 3A). The molecular mechanisms through which RBM15:MKL1 drives AMKL transformation still need to be better understood. Normal functions of RBM15 and MKL1 provide some clues to this fusion’s roles in the AMKL development.

RBM15 is a crucial regulator of N6-methyladenosine methylation of RNA (Zhao et al., 2022). It contains three RNA recognition motifs (RRMs) and a SPEN paralogue and orthologue C-terminal (SPOC) domain (Figure 3A) (Raffel et al., 2007; Niu et al., 2009; Patil et al., 2016; Jin et al., 2018; Schuschel et al., 2020). RBM15 can regulate RNA splicing and histone modification of MPL critical for the HSC and megakaryocyte function (Xiao et al., 2015). RMB15 modulates several transcription factors associated with megakaryocytic differentiation, including RUNX1 (runt-related transcription factor 1), GATA1, and c-MYC (Niu et al., 2009; Zhang et al., 2015). The depletion of RBM15 in the human megakaryoblastic leukemia cell line Meg-01 enhances the formation of alternatively spliced isoforms of RUNX1a and GATA1s (Zhang et al., 2015). RUNX1a (also known as AML1a), a C-terminally truncated RUNX1 isoform, increases DNA binding and affects target gene transcription, with its overexpression increasing functional HSCs and decreasing hematopoietic differentiation (Davis et al., 2021). GATA1s, an N-terminally truncated GATA1 isoform, plays a major role in transient abnormal myelopoiesis and ML-DS development by promoting megakaryocytic progenitor expansion and disrupting megakaryocytic and erythroid differentiation (Wechsler et al., 2002; Shimizu et al., 2009; Chlon et al., 2015; Banno et al., 2016; Juban et al., 2021). The c-MYC proto-oncogene was discovered as a target of RBM15 during HSC and megakaryocyte development (Niu et al., 2009). RBM15 is also involved in regulating the activity of RBPJ (recombination signal binding protein for immunoglobulin kappa J region), which is an important player in the Notch pathway (Ma et al., 2007). *RBM15* overexpression suppresses myeloid differentiation, while its knockdown enhances myeloid differentiation in the myeloid precursor cell line 32DWT18 (Ma et al., 2007). In human umbilical cord blood CD34^+^ cells, *RBM15* knockdown inhibits the maturation of megakaryocytes (Jin et al., 2018). *RBM15* deletion is lethal in embryonic mice, while conditional-knockout causes pleiotropic effects in stem cells and progenitors, including megakaryocytic expansion in the bone marrow and spleen (Raffel et al., 2007).

MKL1, a transcriptional coactivator of serum response factor (SRF), has two isoforms consisting of two or three N-terminal RPEL-repeats, basic regions, a glutamine-rich domain, a SAP domain (homology domain found in SAF-A/B, acinus, PIAS), a leucine zipper-like domain, and a TAD domain (Figure 3A) (Scharenberg et al., 2010; Kalita et al., 2012). MKL1 regulates cell morphology, adhesion, migration and differentiation in various cell types, including myeloid cells (Reed et al., 2021; Sprenkeler et al., 2021; Tabuchi and Ihara, 2021). Beyond being a coactivator for SRF, MKL1 modulates certain transcription factors (e.g., SMADs) and impacts histone modifications (Reed et al., 2021). *MKL1* knockout mice show partial embryonic lethality and aberrant megakaryopoiesis characterized by increased progenitor numbers and reduced numbers of mature megakaryocytes (Sun et al., 2006; Cheng et al., 2009). The ability of MKL1 to promote megakaryocytic maturation largely depends on the SRF regulatory axis. Knockout of either *MKL1* or *SRF* reduces megakaryocytic maturation in primary cultures, while *MKL1* overexpression promotes megakaryopoiesis by augmenting both the genomic associations and activity of SRF (Rahman et al., 2018).

The RBM15::MKL1 fusion protein encompasses all putative functional domains encoded by both genes. Thus the fusion possesses the function of both proteins, including the ability to constitutively activate RBPJ, MKL1-and SRF- dependent target genes (Figure 3A) (Descot et al., 2008; Mercher et al., 2009). In a conditional knockin mouse model, *RBM15::MKL1* causes abnormal megakaryopoiesis during embryonic and adult development but rarely generates AMKL (Mercher et al., 2009). However, in combination with a *MPL* mutation, *RBM15::MKL1* leads to rapid cell transformation and a fatal disease with features similar to human AMKL (Mercher et al., 2009). This suggests that cooperating mutations are required to develop leukemia in the presence of *RBM15::MKL1*. One mechanism through which *RBM15::MKL1* induces leukemia *in vitro* and *in vivo* is through aberrant binding and activation of RBPJ, which is essential for the differentiation bias toward megakaryocytes and proliferation of leukemic cells (Mercher et al., 2009). *RBM15::MKL1* may also exert its oncogenic functions through dysregulation of MKL1 and SRF target genes caused by overactivation of MKL1-and SRF-dependent gene transcription (Descot et al., 2008). *RBM15::MKL1* is associated with histone modifications, which may lead to the epigenetic deregulation of genes that control megakaryopoiesis (Lee and Skalnik, 2012). Levels of *RBM15::MKL1* expression and its endogenous components may also contribute to leukemogenesis. For example, *RBM15::MKL1* overexpression decreases endogenous *RBM15* levels and increases endogenous *MKL1* expression in the megakaryoblastic leukemia cell line 6133, while *RBM15* overexpression reduces the fusion protein expression (Lee and Skalnik, 2012). It seems the N-terminal domain of RBM15 controls endogenous RBM15 expression, but the exact mechanism of this regulation is unknown (Lee and Skalnik, 2012). Relevant hPSC-based human models have been developed to study the impact of *RBM15::MKL1*. These models recreate AMKL features seen in patients, including overexpression of adhesion molecules CDH2 (cadherin 2) and ITGB1, and several components of Notch signaling (Ayllon et al., 2017).

In summary, *RBM15::MKL1* is almost exclusively seen in infants or young children (age <3 years) with AMKL. RBM15::MKL1 possesses the function of both RBM15 and MKL1 proteins, including constitutive activation of RBPJ, MKL1-and SRF- dependent target genes. By itself, *RBM15::MKL1* appears insufficient to drive AMKL. However, with a *MPL* mutation, *RBM15::MKL1* causes rapid transformation to AMKL. It remains to be determined how *RBM15::MKL1* alters megakaryopoiesis and the mechanism through which cooperating mutations drive leukemogenesis.

### 2.5 *HOX* rearrangements

*HOX* rearrangements (*HOX*r) are seen in 14.9% of pediatric non-DS AMKL and associate with better outcomes (5-year overall survival of 77%) than the aforementioned molecular subgroups (*CBFA2T3::GLIS2*, *KMT2A*r, *NUP98::KDM5A*, and *RBM15::MKL1*) (de Rooij et al., 2017). The median age at diagnosis for these patients is 1.5 years (range of approximately 6 months to 2 years) (de Rooij et al., 2017; Masetti et al., 2019b). There are a variety of *HOX*r in AMKL, including *GATA2::HOXA9*, *GATA2::HOXA10*, *NIPBL* (*Drosophila melanogaster* Nipped-B)*::HOXA9*, *NIPBL::HOXB9*, *GATA2::HOXA10*, *EWSR1* (Ewing Sarcoma breakpoint region 1)*::HOXB8*, P*LEK* (pleckstrin)*::HOXA11AS* (HOXA11 antisense RNA), *BMP2K* (BMP-2-inducible protein kinase)*::HOXD10*, *EP300::HOXA7*, *C8orf76* (C8orf76, chromosome 8 open reading frame 76)*::HOXA11AS*, *HOXA11::BZW2* (basic leucine zipper and W2 domains 2), *HOXA9::ANGPT1* (angiopoietin 1) and *HOXA10AS::CD164* (sialomucin core protein 24) (Figure 3B) (de Rooij et al., 2017; Masetti et al., 2019b). *HOX*r cause the upregulation of the *HOX* gene in the fusion and of the adjacent *HOX* genes (de Rooij et al., 2017). As highlighted in previous sections, overexpression of *HOX* genes is also associated with *KMT2A*r and *NUP98::KDM5A*, so together, increased expression of *HOX* genes occurs in approximately half of pediatric non-DS AMKL (de Rooij et al., 2017). Multiple other mutations can be seen in association with *HOX*r in AMKL, including in *NRAS*, *KRAS*, *MPL*, *JAK2*, *PI3K3R1*, *PTEN* (phosphatase and tensin homolog deleted on chromosome 10), *STAG2*, *CTCF* (CCCTC-binding factor), and other genes (e.g., *RB1*, *RUNX1*, *SETX* (senataxin), *ATM* (ataxia-telangiesctasia mutated), *SMARCA2* (SWI/SNF related, matrix associated, actin dependent regulator of chromatin, subfamily A, member 2), *NSD1*, and *TP53*) (de Rooij et al., 2017). *HOX*r AMKL is enriched in *MPL* mutations (41.7%, n = 12) (de Rooij et al., 2017). Murine bone marrow cells transfected with *MPL* p.W515L and *GATA2::HOXA9* or *NIPBL::HOXB9* show growth advantage and increased phosphorylation of JAK2 and STAT5 (de Rooij et al., 2017). Additional cytogenetic aberrations observed in *HOX*r AMKL include gains or losses of certain chromosomes, mostly +5, +6, +8, +10, +16, +18, +19, +21, −6, −7, −8, −16, −17, −19, −22, hyperdiploidy, or a complex karyotype (de Rooij et al., 2017). Given that *HOX*r AMKL is a rare and newly identified entity, little is known about the clinical and laboratory features of patients with this disease subgroup.

Homeobox genes encode a family of homeodomain-containing transcription factors that play diverse roles ranging from embryogenesis to carcinogenesis, including hematopoiesis and leukemogenesis (Bhatlekar et al., 2018; Collins and Thompson, 2018). In humans, a total of 39 *HOX* genes are found, situated in clusters on four chromosomes (7p15, 17q21.2, 12q13, and 2q31) (Bhatlekar et al., 2018; Collins and Thompson, 2018). *HOX* genes are grouped as *HOXA*, *HOXB*, *HOXC*, and *HOXD* (Figure 3B). Except for HOXD, HOX transcription factors play essential roles in the generation of blood cells (Bhatlekar et al., 2018; Collins and Thompson, 2018). For example, HOXA9, one of the most abundant HOX transcription factors in HSCs, impacts HSC proliferation, self-renewal, and myeloid and lymphoid differentiation (Bhatlekar et al., 2018; Collins and Thompson, 2018). *HOXA9* is overexpressed in more than 50% of AML, which is associated with poor outcomes (Andreeff et al., 2008; Rio-Machin et al., 2017; Gavory et al., 2021). A recurrent fusion of *NUP98* with *HOXA9* via chromosome translocation t(7;11)(p15;p15) drives AML by deregulating expression of *MEIS1*, *HOXA9*, and *PBX3*, arresting differentiation and inducing long-term proliferation of human HSCs (Takeda et al., 2006; Rio-Machin et al., 2017). Nevertheless, this fusion has not been associated with the megakaryocytic phenotype. In contrast, *GATA2::HOXA9* and *NIPBL::HOXB9* have been demonstrated to efficiently generate AMKL in mice (Dang et al., 2017). Both fusions transfected into murine hematopoietic cells upregulate RUNX1, FLI1, and HOX target genes, increase the self-renewal capacity of HSCs and cause dysplastic megakaryopoiesis (Dang et al., 2017). *HOX*r are predicted to produce either in-frame functional fusion proteins or loss of function of the regulatory transcripts, highlighting the crucial roles of *HOX* genes in the pathogenesis of pediatric non-DS AMKL (de Rooij et al., 2017). However, more studies are required on the mechanism through which *HOX*r drive AMKL.

### 2.6 Non-recurrent gene fusions and unknown drivers

A few non-recurrent fusion genes have been identified in 3.5% of pediatric non-DS AMKL, including *MN1::FLI1*, *GRB10* (growth factor receptor bound protein 10)*::SDK1* (sidekick cell adhesion molecule 1), *BCR::ABL1* and *MAP2K2::AF10* (de Rooij et al., 2017). *MN1::FLI1* induces AMKL in mice with a strong gene expression signature characteristic of megakaryocyte progenitors (Dang et al., 2017). Chimeric transcripts harboring *STAG2* (e.g., *STAG2::GPR119* (G protein-coupled receptor 119) and *STAG2::LINCO1285*) are detected in 3.5% of non-DS AMKL, and all are predicted to induce a truncated STAG2 protein (de Rooij et al., 2017). Because of the very low incidence of non-recurrent gene fusions in AMKL, little is known about their roles in leukemogenesis.

Monosomy 7 is detected in 5%–6% of pediatric AMKL and may confer poor risk (de Rooij et al., 2016; de Rooij et al., 2017) (Supplementary Table S1). Monosomy 7 is common in *CBFA2T3::GLIS2*, *HOX*r, and unknown driver subgroups (de Rooij et al., 2016; de Rooij et al., 2017). However, how monosomy 7 contributes to AMKL pathogenesis remains poorly understood.

Finally, the aetiology of approximately 15% of pediatric AMKL without any known genetic aberrations is unknown (de Rooij et al., 2017). Therefore, other molecular drivers are expected to be found.

### 2.7 *GATA1* mutations

Although truncating mutations in *GATA1* exon 2 or 3 are known to play essential roles in ML-DS, they can also be found in 9%–10% of pediatric non-DS AMKL (Figure 1A) (de Rooij et al., 2017). The non-DS AMKL patients with *GATA1* mutations do not have germline trisomy 21 or any physical stigmata of DS, but *GATA1* mutations and chromosome 21 amplifications are observed in major leukemic clones in these patients (de Rooij et al., 2017). Amplifications of chromosome 21 are one of the most highly recurrent copy number alterations found in approximately 39% of children with non-DS AMKL, possibly because certain chromosome 21 genes (e.g., *DYRK1A* [dual specificity tyrosine phosphorylation regulated kinase 1A] and *ERG*) act as critical players in megakaryopoiesis (de Rooij et al., 2017; Li and Kalev-Zylinska, 2022). Like ML-DS, *GATA1* mutant cases of non-DS AMKL show significant overexpression of chromosome 21 genes and co-operating mutations in cohesins and the JAK pathway. Also similar to ML-DS, these patients have excellent outcomes (de Rooij et al., 2016; de Rooij et al., 2017; Masetti et al., 2019b) (Figure 1B). Because of these crucial similarities with ML-DS, non-DS AMKL cases with somatic *GATA1* mutations are referred to as DS-*like* AMKL (de Rooij et al., 2017).

## 3 Novel molecularly targeted therapeutic strategies being developed for pediatric non-DS AMKL

In pediatric non-DS AMKL, high-risk subgroups (*CBFA2T3::GLIS2*, *KMT2A*r, and *NUP98::KDM5A*) have inferior outcomes, mostly because of the primary refractoriness to chemotherapy and/or early relapse (de Rooij et al., 2016; de Rooij et al., 2017). Novel therapeutic strategies are urgently needed to improve the long-term survival of these patients. With the molecular abnormalities of pediatric non-DS AMKL being unravelled, some novel molecular targets are being considered, including driver fusions, their associated molecules, downstream targets or signaling pathways, and cooperating alterations or their related molecules (Table 3). Next, we present a brief overview of selected therapeutic targets being developed for AMKL.

**TABLE 3:** Novel molecular targets/drugs being considered in high-risk pediatric non-DS AMKL.

Molecular category/molecular target	*CBFA2T3::GLIS2*	*KMT2A*r	*NUP98::KDM5A*
Fusion protein	GLIS2 (Masetti et al., 2017)		KDM5A (PHD domain) (Michmerhuizen et al., 2020)
Protein-protein interactions		Interaction between KMT2A and menin (Krivtsov et al., 2019; Klossowski et al., 2020); Interaction between KMT2A fusion partners and DOT1 (Yi and Ge, 2022)	Interaction between KMT2A and menin (Heikamp et al., 2022)
Downstream molecules or pathways	CD56 (Smith et al., 2020); Super enhancer, KIT and PDGFRA (Benbarche et al., 2022); FOLR1 (Le et al., 2022); BCL2 family members (e.g., BCL2, MCL1, BCL-xL) (Aid et al., 2023; Kuusanmaki et al., 2023)	CDK6 (Placke et al., 2014; Schmoellerl et al., 2020); HOXA (de Rooij et al., 2017)	CDK6 (Schmoellerl et al., 2020); JAK (Cardin et al., 2019; Noort et al., 2021); HOXA and HOXB (Noort et al., 2021)
Co-occurring mutations		RAS mutations (de Rooij et al., 2017; Mansur et al., 2017; Chu et al., 2018)	RB1 loss (de Rooij et al., 2017)
Other potential targets	Aurora A (Thiollier et al., 2012)	IKAROS (Aubrey et al., 2022); LIM (Jensen et al., 2020); HOXA10-AS (Al-Kershi et al., 2019); MEK, PI3K (Lopes et al., 2022); MYC, BCL2, SIRT1, BRD4, LSD1, DNMT, CDK9 (Yi and Ge, 2022)	Aurora A (Masetti et al., 2019b); MEK, tubulin, PI3K, BRD4, CDK9, HSP90 (Noort et al., 2021)
Examples of drug combinations including targeted and chemotherapeutic agents in clinical use		CD33 targeted agent + anthracycline and cytarabine-based chemotherapy (tested in patients with *KMT2A*r AML, subtype unspecified) (Pollard et al., 2021); DOT1L inhibitor + standard chemotherapy (NCT03724084) (tested in patients with newly diagnosed *KMT2A*r AML, subtype unspecified) (Yi and Ge, 2022); DOT1L inhibitor + DNMT inhibitor (NCT03701295) (tested in patients with relapsed, refractory, or newly diagnosed *KMT2A*r AML, subtype unspecified) (Yi and Ge, 2022)	Bromodomain inhibitors + gemcitabine (tested in preclinical models of *NUP98-KDM5A* erythroleukemia) (Iacobucci et al., 2021)
Examples of pre-clinical agents that target disease-associated molecular alterations and deregulated signaling pathways	BCL2 inhibitor + MCL1 inhibitor (tested in AMKL and non-AMKL AML models) (Aid et al., 2023); BCL-xL inhibitor + JAK inhibitor (tested in AMKL and non-AMKL AML models) (Kuusanmaki et al., 2023)	Menin inhibitor + DOT1L inhibitor (tested in *KMT2A*r non-AMKL models including AML and ALL) (Dafflon et al., 2017); Menin inhibitor + IKAROS degradation (immunomodulatory imide drugs) (tested in *KMT2A*r or *NPM1* AML models, subtype unspecified) (Aubrey et al., 2022); Menin inhibitor + BCL2 inhibitor (tested in *KMT2A*r or *NPM1* AML models, subtype unspecified) (Fiskus et al., 2022); Menin inhibitor + CDK6 inhibitor (tested in *KMT2A*r or *NPM1* AML, subtype unspecified) (Fiskus et al., 2022); Menin inhibitor + FLT3 inhibitor (tested in non-AMKL cell lines harboring both *KMT2A*r and FLT3-ITD mutations) (Miao et al., 2020); Menin inhibitor + DHODH inhibitor (tested in *KMT2A*r non-AMKL model) (Brzezinka et al., 2019); DOT1L inhibitor + SIRT1 activator (tested in *KMT2A*r non-AMKL leukemia, including AML and ALL) (Chen et al., 2015); DOT1L inhibitor + LSD1 inhibitor (tested in *KMT2A*r non-AMKL models) (Feng et al., 2016); DOT1L inhibitor + CDK9 inhibitor (tested in *KMT2A*r non-AMKL leukemia) (Brzezinka et al., 2019)	

The aberrant expression of fusion proteins in leukemic cells represents an opportunity for therapeutic targeting and is of significant research interest. For example, GANT61 is a small molecule that represses the DNA-binding and transcriptional activities of GLI proteins (Masetti et al., 2017; Lau et al., 2019). Since GLIS2 has a high homology of DNA-binding domain with other GLI proteins, GANT61 may target CBFA2T3:GLIS2 fusion in pediatric AML (Masetti et al., 2017; Lau et al., 2019). *CBFA2T3::GLIS2* positive non-AMKL and AMKL cell lines (WSU-AML and M07e, respectively) and primary leukemic cells from patients are more sensitive to GANT61 than fusion-negative cells (Masetti et al., 2017). GANT61 induces apoptosis and G1 cell-cycle arrest, decreases the expression of *GLIS2*, *CBFA2T3::GLIS2* and its target molecules (e.g., CD56, GATA3, CRISP3, and H2AFY) (Masetti et al., 2017). Nevertheless, further testing of GANT61 in mice models of *CBFA2T3::GLIS2* leukemia is required.

Targeting interactions of the driver fusion with its associated molecules is another way to inhibit its leukemogenic drive. For instance, KMT2A directly binds to menin through the MBD domain and forms a menin-KMT2A complex. This complex is critical in regulating the *HOX* gene cluster, including the leukemogenic *HOXA9* and its co-factor *MEIS1* in myeloid stem and progenitor cells (Li and Song, 2021; Fiskus et al., 2022). Hence, disrupting the interaction between KMT2A and menin has emerged as a promising strategy. Menin inhibitors (e.g., VPT-50469, MI-3454, KO-539, and SNDX-5613) are capable of perturbing menin binding to KMT2A fusion, leading to a reduction in expression of its key targets, such as HOXA9, MEIS1, FLT3, and CDK6 (Krivtsov et al., 2019; Klossowski et al., 2020; Li and Song, 2021). Menin inhibitors inhibit proliferation and promote differentiation and apoptosis in non-AMKL cell lines expressing *KMT2A*r, and achieve remission in *KMT2A*r non-AMKL mouse models, including patient-derived xenografts (Krivtsov et al., 2019; Klossowski et al., 2020; Li and Song, 2021). Two inhibitors KO-539 and SNDX-5613 have entered phase I/II clinical trials for treating refractory/relapsed *KMT2A*r AML (NCT04067336 and NCT04065399) (Li and Song, 2021). Leukemic cells with *NUP98* fusions are also dependent on KMT2A, as KMT2A recruits a fusion onto the *HOXA* gene locus. Inhibition of interactions between KMT2A and menin by VTP50469 upregulates expression of molecules required for megakaryocytic and erythroid differentiation and downregulates expression of pro-leukemogenic genes (e.g., *HOXA* cluster) (Heikamp et al., 2022). VTP50469 prolongs the survival of mice engrafted with *NUP98::KDM5A* patient-derived cells (Heikamp et al., 2022). These findings highlight that targeting KMT2A-menin interactions may be helpful as a novel therapeutic approach. Likewise, targeting interactions between KMTA2 fusion partners and DOT1L emerged as a valid strategy against *KMT2A*r leukemia. DOT1L peptide mimetics and/or small molecule inhibitors disrupting interactions between DOT1L and AF9/AF10/ENL are currently under investigation (Wu et al., 2021; Yi and Ge, 2022; Yuan et al., 2022). For example, a DOT1L peptide mimetic was synthesized to target DOT1L and AF9/ENL, and its use supressed growth of non-AMKL leukemic cell lines harboring *KMT2A*r (Yuan et al., 2022). The most potent mimetic has similar anticancer activities to the DOT1L inhibitor EPZ5676 in *KMT2*Ar non-AMKL cell lines, demonstrating that inhibition of interactions between DOT1L and a KMT2A fusion is a promising approach (Yuan et al., 2022). DOT1L inhibitor EPZ5676 was trialled in pediatric and adult patients with *KMT2A*r refractory or relapsed leukemia (NCT02141828 and NCT01684150 clinical trials) (Yi and Ge, 2022). The drug was well tolerated but had modest clinical activity due to drug resistance emerging upon long-term administration (Yi and Ge, 2022). It was concluded that EPZ5676 might be more efficacious when used in synergy with other anti-leukemic agents. For instance, a combination of DOT1L inhibitor with an inhibitor of the KMT2A-menin interactions was proposed to overcome resistance to a single agent (Dafflon et al., 2017).

Targeting downstream targets or signaling pathways of the driver fusion represents another rational approach to designing anti-leukemic treatment. For example, *CBFA2T3::GLIS2* is associated with higher expression of CD56 and FOLR1, and activation of super enhancers, which provides highly plausible therapeutic targets (Benbarche et al., 2022). Surface expression of CD56 is high in patient-derived blasts, and an anti-CD56 antibody-drug conjugate (m906-PBD-ADC) exhibits a CD56-specific cell killing against primary leukemic blasts carrying this fusion (Smith et al., 2020). FOLR1 has also been validated as a valuable target in *CBFA2T3::GLIS2* AMKL (Le et al., 2022). CAR T cells against FOLR1 showed pre-clinical efficacy in leukemic cell lines, patient-derived cells and xenografts of *CBFA2T3::GLIS2* leukemia (Le et al., 2022). One super enhancer specific for leukemic cells carrying *CBFA2T3::GLIS2* controls the expression of KIT and PDGFRA (Benbarche et al., 2022). Inhibition of this super enhancer combined with tyrosine kinase inhibitors specific for KIT and PDGFRA are able to impair leukemic progression in xenograft models, validating super enhancers, KIT and PDGFRA as useful therapeutic targets (Benbarche et al., 2022). Other targets are also being investigated. High expression of STAT5 in AMKL cell lines correlates with sensitivity to JAK inhibitor ruxolitinib (Drenberg et al., 2019). In three distinct murine models of AMKL carrying *CBFA2T3::GLIS2* alone, in combination with *JAK2* p.V617F, or with copy number alterations on chromosome 21, ruxolitinib significantly prolongs survival, justifying its therapeutic testing in pediatric AMKL (Drenberg et al., 2019). A new study showed that patient-derived *CBFA2T3::GLIS2*-positive AMKL cells express high levels of pro-apoptotic *CASP3* and anti-apoptotic *BCL2* (Aid et al., 2023). *CBFA2T3::GLIS2*-positive cell lines are dependent on BCL2 family members for cell survival. Combined targeting of BCL2 (using ABT199) and the myeloid cell lymphoma-1 (MCL1) protein (using *S63845*) inhibits the proliferation of *CBFA2T3::GLIS2* cells *in vitro* and abrogates leukemia progression in mouse xenografts (Aid et al., 2023). Another pro-survival protein BCL-xL was also identified as a potential therapeutic vulnerability in erythroid and megakaryocytic lineage leukemias, including in the M07e AMKL cell line harboring *CBFA2T3::GLIS2* (Kuusanmaki et al., 2023). The BCL-xL-specific inhibitor A-1331852 inhibits growth of erythroid and megakaryocytic blasts, patient-derived and cell lines, including cell lines resistant to BCL2 inhibitor venetoclax. The combination of A-1331852 with ruxolitinib eliminates growth of the erythroid (TF1 and HEL) and megakaryocytic (CMK) cell lines in long-term cultures spanning over a month (Kuusanmaki et al., 2023). These findings suggest new therapeutic targets for further testing in AMKL and reinforce the value of retaining the cell of origin information in AML classification (Arber et al., 2022; Khoury et al., 2022; Brown and Wei, 2023).

In *NUP98::KDM5A*-positive patient cells and mouse models, CDK6 and JAK-STAT pathways are upregulated (Cardin et al., 2019; Schmoellerl et al., 2020). Studies show that *NUP98::KDM5A*-positive cells are sensitive to CDK4/6 inhibitor (palbociclib) and JAK inhibitors (ruxolitinib and tofacitinib) (Cardin et al., 2019; Schmoellerl et al., 2020). A combination of menin inhibitor SNDX-50469 and CDK6 inhibitor abemaciclib has a synergistic activity in non-AMKL cell lines (MOLM13 and MV4-11) and patient-derived AML blasts harboring *KMT2A*r (Fiskus et al., 2022).

Targeting cooperating alterations or their related molecules may assist in leukemia treatment. For example, MEK (mitogen-activated protein kinase) inhibitors (selumetinib and MEK162) have been proposed as potential options for *KMT2A*r infants with ALL carrying *RAS* mutations (Kerstjens et al., 2017; Mansur et al., 2017; Chu et al., 2018). *RAS* mutations commonly occur in *KMT2A*r AMKL (de Rooij et al., 2017); thus inhibition of the RAS pathway may be helpful in this disease, but this awaits experimental testing. Other possible therapeutic targets in three molecular subtypes of AMKL classified into high-risk category include Aurora A, PI3K, BRD4 (bromodomain-containing protein 4), CDK9 (cyclin-dependent kinase 9), HSP90 (heat shock protein 90), CD33, IKAROS, LIM (Lin11/Isl1/Mec3), HOXA10-AS, SIRT1 (sirtuin 1), LSD1 (lysine-specific demethylase 1), DNMT, c-MYC, BCL-2 (B Cell lymphoma 2), and ATM (Thiollier et al., 2012; Al-Kershi et al., 2019; Jensen et al., 2020; Smith et al., 2020; Noort et al., 2021; Pollard et al., 2021; Aubrey et al., 2022; Lopes et al., 2022; Yi and Ge, 2022). Foretinib (GSK1363089) is an oral multikinase inhibitor targeting MET (mesenchymal-epithelial transition factor), RON (recepteur d’origine nantais), AXL (AXL receptor tyrosine kinase), VEGFR (vascular endothelial growth factor receptor), c-KIT, FLT3, and PDGFR pathways. Patient-derived non-AMKL leukemic cells containing *KMT2A*r and FLT3 mutations have a higher sensitivity to foretinib, suggesting this drug could benefit patients with multiple molecular aberrations (Lopes et al., 2022).

Rational combination therapies will be essential to improve treatment for high-risk AMKL patients. Combined therapies may include conventional cytotoxic drugs integrated with molecularly targeted agents or combinations of targeted agents informed by molecular alterations present in patients. The addition of anti-CD33 antibody gemtuzumab ozogamicin to conventional chemotherapy improves outcomes of children with *KMT2A*r AML (Pollard et al., 2021). Another pre-clinical example is that menin inhibitor SNDX-5613 combined with BCL2 inhibitor venetoclax shows synergistic activity in patient-derived *KMT2A*r AML cells and PDX mouse models (Fiskus et al., 2022). Table 3 contains examples of drug combinations tested in different experimental models and/or clinical trials against leukemias driven by *CBFA2T3::GLIS2*, *NUP98::KDM5A*, and *KMT2A*r.

## 4 Conclusion

Pediatric non-DS AMKL is a rare, heterogeneous entity characterized by mostly poor outcomes, in contrast to excellent outcomes seen in ML-DS. Assisted by advanced genomic characterization, pediatric non-DS AMKL has been divided into distinct molecular subtypes. In the latest 2022 WHO and ICC classifications, cases with *KMT2A* and *NUP98* rearrangements form independent AML subgroups, and cases with *CBFA2T3::GLIS2* and *RBM15::MKL1* belong to a subgroup of AML with other defined genetic alterations. Patients with *CBFA2T3::GLIS2*, *KMT2A*r and *NUP98::KDM5A* have adverse risk, while other AMKL patients (e.g., with *RBM15::MKL1* and *HOX*r) are considered an intermediate risk, except for DS-like AMKL that has an excellent prognosis. Different subgroups of AMKL share similarities and differences in gene and transcript changes, which offer new therapeutic targets. Novel drugs that interfere with driver fusions, downstream molecules, and cooperating alterations are being developed for these patients.

Progress in understanding AMKL pathogenesis has been immense in the last decade, but many challenges persist. We still need to improve our knowledge of this disease’s genetic and molecular landscape. Leukemogenic drivers are unknown in approximately 15% of non-DS AMKL, and the role of many known genetic alterations and cooperating events is unclear. Further progress in these areas will be critical to enable better insights into AMKL pathogenesis and improve patient outcomes.
