# Supplementary material for: Advances in molecular characterization of pediatric acute megakaryoblastic leukemia not associated with Down syndrome; impact on therapy development
Source: Front Cell Dev Biol. 2023 Jun 1;11:1170622. doi: 10.3389/fcell.2023.1170622 (PMC10267407; doi:10.3389/fcell.2023.1170622)
Supplement: Supplementary file 1 [file Table1.pdf]

Supplementary Table S1. Key features of AMKL associated with monosomy 7 and all other types of pediatric non-DS AMKL combined.

Molecular category/ characteristics	AMKL associated with monosomy 7	Non-DS AMKL (all combined)
Median age at diagnosis, years (range) (n)	0.7 (0.5-1.5) (n=5) (de Rooij et al., 2017)<br>1.5 (0.5-17.1) (n=9) (de Rooij et al., 2016)	1.4 (0.1-12.2) (n=87) (de Rooij et al., 2017)<br>1.6 (0.1-17.1) (n=153) (de Rooij et al., 2016)<br>1 (0-13) (n=44) (Hara et al., 2017)<br>1.9 (1.4-11) (n=6) (Lalonde et al., 2021)
Male, % (n)	40 (n=5) (de Rooij et al., 2017)<br>22 (n=9) (de Rooij et al., 2016)	52 (n=87) (de Rooij et al., 2017)<br>46 (n=153) (de Rooij et al., 2016)<br>48 (n=44) (Hara et al., 2017)<br>67 (n=6) (Lalonde et al., 2021)
Median white cell count, $\times 10^9$/L (range) (n)	14.5 (8.7-90.2) (n=9) (de Rooij et al., 2016)	13.7 (1.1-378.5) (n=153) (de Rooij et al., 2016)<br>22 (4.3-191.6) (n=44) (Hara et al., 2017)
Additional chromosomal abnormalities (%) (n)	Complex (80%, n=5) (de Rooij et al., 2017)	Complex (52%), +21 (39%), +19 (24%), +6 (9%), +8 (8%), -19 (6%), +14 (5%), -7 (5%), -16 (5%), del(9q) (5%), del(13q) (5%), +2 (3%), -10 (3%), del(6q) (3%), del(5p) (2%), del(9p) (2%), add(5p) (2%), add (16q) (2%) (n=87 for all) (de Rooij et al., 2017)<br>Hyperdiploidy (52%), complex (50%), +21 (36%), -7 (7%) (n=44 for all) (Hara et al., 2017)<br>Complex (50%), +21 (33%) (n=6 for both) (Lalonde et al., 2021)
Associated mutations (%) (n)		MPL (14%), JAK1 (3%), JAK2 (9%), JAK3 (5%), RB1 (14%), GATA1 (12%), NRAS (9%), KRAS (7%), PTPN11 (3%), CTCF (8%), STAG2 (5%), SMC1A (3%), RAD21 (2%), PIK3C2A (5%), PIK3CA (2%), PIK3R1 (2%), BCOR (2%), TP53 (2%) (n=87 for all) (de Rooij et al., 2017)<br>GATA1 (11%), FLT3-ITD (9%), NRAS (7%), KIT (7%), WT1 (5%) (n=44 for all) (Hara et al., 2017)<br>SETD2 (33%) (n=6) (Lalonde et al., 2021)
4-year EFS rate, % (n)	33±16 (n=9) (de Rooij et al., 2016)	51±4 (n=153) (de Rooij et al., 2016)<br>37 (n=44) (Hara et al., 2017)
4-year OS rate, % (n) except where indicated	33±16 (n=9) (de Rooij et al., 2016)	56±4 (n=153) (de Rooij et al., 2016)<br>59 (n=44) (Hara et al., 2017)<br>5-year: 59 (n=44) (Smith et al., 2020)

n, number of patients in different studies; EFS, event-free survival; OS, overall survival.
